# Genetic variations on 31 and 450 residues of influenza A nucleoprotein affect viral replication and translation

**DOI:** 10.1186/s12929-019-0612-z

**Published:** 2020-01-06

**Authors:** Su-Jhen Hung, Yin-Mei Hsu, Sheng-Wen Huang, Huey-Pin Tsai, Leo Yi Yang Lee, Aeron C. Hurt, Ian G. Barr, Shin-Ru Shih, Jen-Ren Wang

**Affiliations:** 1 Department of Medical Laboratory Science and Biotechnology, College of Medicine, National Cheng Kung University, No.1, University Road, Tainan, 701 Taiwan; 2 National Mosquito-Borne Diseases Control Research Center, National Health Research Institutes, Tainan, Taiwan; 3 Department of Pathology, National Cheng Kung University Hospital, Tainan, Taiwan; 4 WHO Collaborating Centre for Reference and Research on Influenza, Victorian Infectious Diseases Reference Laboratory, Peter Doherty Institute for Infection and Immunity, Melbourne, Victoria 3000 Australia; 5 Department of Medical Biotechnology and Laboratory Sciences, College of Medicine, Chang Gung University, Taoyuan, Taiwan; 6 Center of Infectious Disease and Signaling Research, National Cheng Kung University, Tainan, Taiwan; 7 National Institute of Infectious Diseases and Vaccinology, National Health Research Institutes, Tainan, Taiwan

**Keywords:** Influenza virus, Nucleoprotein, Evolution, H3N2, Ferret study, Viral replication, Viral translation

## Abstract

**Background:**

Influenza A viruses cause epidemics/severe pandemics that pose a great global health threat. Among eight viral RNA segments, the multiple functions of nucleoprotein (NP) play important roles in viral replication and transcription.

**Methods:**

To understand how NP contributes to the virus evolution, we analyzed the NP gene of H3N2 viruses in Taiwan and 14,220 NP sequences collected from Influenza Research Database. The identified genetic variations were further analyzed by mini-genome assay, virus growth assay, viral RNA and protein expression as well as ferret model to analyze their impacts on viral replication properties.

**Results:**

The NP genetic analysis by Taiwan and global sequences showed similar evolution pattern that the NP backbones changed through time accompanied with specific residue substitutions from 1999 to 2018. Other than the conserved residues, fifteen sporadic substitutions were observed in which the 31R, 377G and 450S showed higher frequency. We found 31R and 450S decreased polymerase activity while the dominant residues (31 K and 450G) had higher activity. The 31 K and 450G showed better viral translation and replication in vitro and in vivo.

**Conclusions:**

These findings indicated variations identified in evolution have roles in modulating viral replication in vitro and in vivo. This study demonstrates that the interaction between variations of NP during virus evolution deserves future attention.

## Background

Influenza A viruses are common respiratory infectious pathogens which cause severe epidemics and occasional pandemics [1]. These pandemic influenza strains then persistently circulate in the human population and cause seasonal epidemics. For example, H3N2 viruses have continuously circulated in human population since the 1968 pandemic. Influenza A viruses contain eight gene segments, which include PB2, PB1, PA, HA, NP, NA, M, and NS [2]. Antigenic shift is caused by reassorting of gene segments while antigenic drift is caused by an accumulation of mutations in genes during virus evolution. Under the pressure of host immunity and the environment, viruses which can survive with new mutations, may keep circulating and potentially become a major public health threat [3]. Consequently, although specific anti-viral drugs as well as vaccination strategies have been developed and applied, annual seasonal epidemics still result in millions of severe cases and hundreds of thousands of deaths according to the World Health Organization (WHO). Thus, it is essential to better understand the evolutionary mechanisms of influenza A viruses.

Replication and transcription of influenza viruses rely on the functional unit, ribonucleoprotein (RNP) complex structure, in which viral RNA is encapsidated by viral polymerase PB2, PB1, PA and NP. In the RNP complex, NP in homo-oligomer form not only stabilizes this structure but also modulates viral RNA transcription and replication through interaction with viral polymerase PB2 and PB1. It has been reported that NP is also crucial in RNA elongation during viral RNA replication [4]. Previous studies have demonstrated that the homo-oligomer formation and RNA binding are crucial for virus replication and a single amino acid mutation can reduce the polymerase activity [5–8]. In regards to function, NP contains an RNA binding domain, a PB2 binding domain, a homo-oligomer binding domain and a nuclear localization signal domain (NLS) [6–10]. Many studies further observed that NP interacts with many host factors to promote viral replication, escape immunity, or regulate apoptosis [11].

Most previous studies on influenza A have focused on the change of viral surface proteins, HA and NA. For instance, A(H3N2) viruses have been monitored for antigenic variants and different vaccine strains have been selected based on their novel antigenic characteristics that can be mapped by hemagglutination inhibition [12], and also identified by their HA gene sequences. However, the evolution of internal genes, such as NP, also plays an important role in viral fitness and pathogenicity, but has generally lacked attention. We hypothesized that in the evolutionary history of a relatively conserved protein, mutations arising over time might also be meaningful. Thus, we evaluated the influenza virus NP, a major multi-functional protein in the virion, to identify novel evolutionary or functionally important determinants of viral replication. Previous studies have shown that the evolution rates of viral genomes in influenza A are different with the rate in H3N2 greater than in H1N1 and also than influenza B viruses [13, 14]. Clinical reports also showed that the H3N2 viruses led to high mortality in the 1991–1998 and 2003–2004 influenza seasons, in which the latter was associated with an unusually high number of fatalities in children. In addition, several studies also demonstrated that the H3N2 caused more severe diseases than H1N1 and influenza B viruses [15–17]. In Taiwan, H3N2 influenza viruses were monitored as the main circulating subtype, its epidemic rate was higher than H1N1, and the HA gene was consistent phylogenetically with H3N2 viruses reported elsewhere [18, 19]. Thus, in our study, clinical isolates of H3N2 viruses that were kept from surveillance samples at the National Cheng Kung University Hospital were used to examine novel determinants on NP that may play a role in influenza virus evolution.

## Methods

### Cell lines and virus isolates

MDCK and A549 cells were cultured in DMEM supplemented with 10% fetal bovine serum (FBS) and 2% Penicillin/Streptomycin (P/S); 293T cells were cultured in DMEM supplemented with 10% FBS, 2% P/S and 1X sodium pyruvate. Clinical isolates of influenza A virus H3N2 subtypes were collected from the Virology Laboratory of National Cheng Kung University Hospital (NCKUH) between 1999 and 2017. Influenza isolates were cultured in MDCK cells; the virus culture medium was DMEM supplemented with 2% (P/S) and 2.5 μg/mL trypsin. MDCK cells were used in virus growth kinetics and plaque assay. A549 cells were also used to analyze virus growth kinetics. 293T cells were used in the transfection assay.

### Sequence analysis of NP gene

Viral RNAs were extracted from cultured viruses by Total RNA Extraction kit (RBC Bioscience). RNAs were reverse transcribed into cDNA and amplified via PCR (KOD Plus kit, Toyobo) using NP specific primer pair AGCAAAAGCAGGGTTAATAA and ATATCGTCTCGTATTAGTAGAAACAAGGGTATTTTT. The reverse transcription was performed at 42 °C for 60 mins and then 94 °C for 5 mins. The PCR reactions were 30 cycles of 94 °C for 30 s, 50 °C for 30 s, and 72 °C for 1.5 mins. NP DNA were purified using Gel/PCR DNA Fragment Extraction kit and sequenced by the Applied Biosystems 3130XL Genetic Analyzer (Center for Genomic Medicine of NCKU).

### Phylogenetic and amino acid substitution analysis

Sequence alignment was operated by the BioEdit software. Reference strains of H3N2 subtype were obtained from the GenBank database. Phylogenetic analysis of our sequences from isolates and reference strains was carried out by the MEGA 7 software. The aligned sequences were translated into amino acid sequences by BioEdit software.

### Analysis of sequences from influenza research database

To expand the evolutional analysis, we collected 14,220 H3N2 NP sequences obtained from the NIAID Influenza Research Database (IRD) (http://www.fludb.org) [20]. In this database, the origin of sequence could be set before download, so we further gated by host (human) and year (from 1999 to 2018; every year in separate), and alignments were done on the IRD website. Downloaded aligned nucleotide sequences were translated to amino acid sequences. The number of every amino acid sequence type in each year was calculated and got the percentage in total sequence number; amino acid sequence types more than 5% were listed in Table 1. Since every year was analyzed independently, we could observe the changes in amino acid sequence types through the year, and the most dominant one could be the same in different years (for example, the most dominant type was the same in 2001, 2002 and 2003).

**Table 1:**
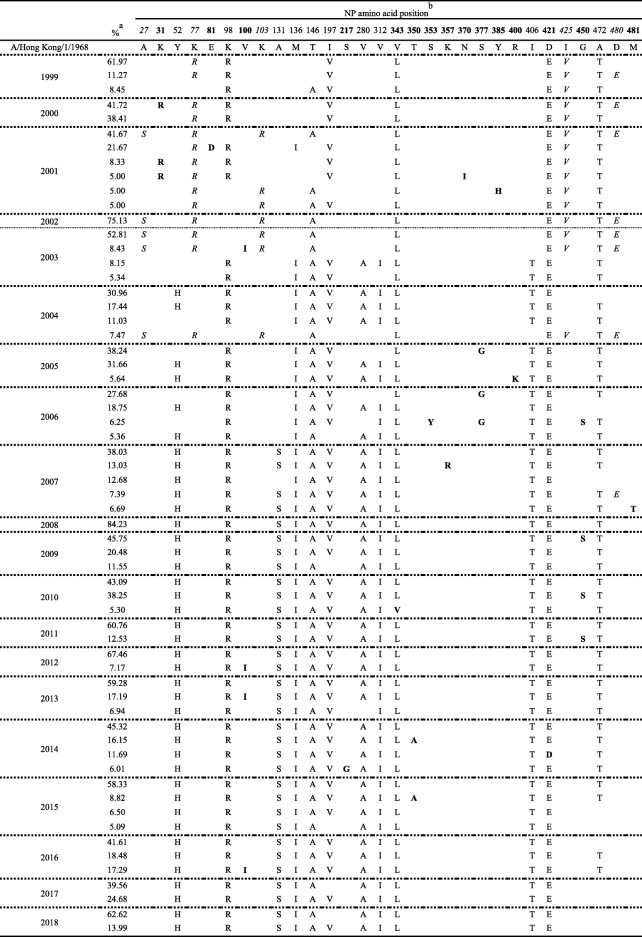
The dominant NP amino acid sequences of each year from 1999 to 2018

^a^The sequence types, their number and the proportion to total sequence collected in the same year were analyzed and calculated. Only percentage over 5% in each year were listed in this table

^b^Residues in italic were those major observed from 1999 to 2003 especially in 2001–2003 and rarely identified after 2004. Residues in normal form were defined as conserved protein backbone especially since 2004 in longer time period while residues in bold were defined as sporadic substitutions during evolution (Please see the description in Results text)

The residues in bold in Table 1 defined as sporadic substitutions because they only existed in dominant types in several years and did not become conserved. The number of any amino acid sequence type with a sporadic substitution was calculated and the percentage in total sequence number was obtained (Fig. 2). Table 1 showed the dominant sequence types with their percentage, while Fig. 2 showed the amount of sporadic substitutions in each year, which may be identified in various sequence types even in those that were not listed in Table 1 (< 5%).

### Construction of NP expression plasmids and site-directed mutagenesis

To evaluate NP genetic variations by mini-genome assay, point mutations were introduced into the NP gene of A/Taiwan/3446/02 strain by site-directed mutagenesis PCR with specific primer pairs. All of the mutations analyzed in Fig. 3 were generated from the A/Taiwan/3446/02 NP backbone. Mutations in Fig. 3ab were arranged from KSG because of the dominant circulating sequence being KSG. The template plasmids were removed by DpnI. *E. coli* containing NP mutation plasmids were amplified in LB broth and plasmids were extracted by Qiagen plasmid midi kit for further analysis.

### Mini-genome assay

For mini-genome assay, 293 T cells were seeded in 96-well plates in the concentration of 2.7 × 10^4^ cells/0.1 mL. After 24 h of culture, cells were transfected with influenza PB2, PB1, PA expression plasmids (A/Taiwan/3446/02 strain in pHW2000 vector) and different NP expression plasmids by Lipofectamine 2000. Dual-luciferase expression plasmids were co-transfected, whereby firefly luciferase acted as the reporter and renilla luciferase acted as the internal control. The reporter plasmid contains 3′ and 5′ noncoding region of the influenza virus and is under the control of Pol-I promoter as well as the Pol-I terminator. Transfected cells were cultured at 33 °C or 37 °C, which resembled the temperature of the human upper and lower respiratory tract respectively. Cell lysates were collected in 0.1 mL Passive lysis buffer. Luciferase activity of cell lysate was analyzed with dual-luciferase assay kit (Promega) and detected by VICTOR 1420 microplate reader.

### Production of reverse genetics viruses

For production of reverse genetics virus, 293 T cells were seeded in 6-well plates in the concentration of 1 × 10^6^ cells/2 mL. After 24 h of culture, cells were transfected with 7 influenza gene expression plasmids (A/Taiwan/3446/02 strain) and different NP expression plasmid by PolyJet reagent. After 24 h, culture medium was changed to serum-free DMEM and then cultured for an additional 48 h. Three days after transfection, the total cell lysates were collected for immunofluorescence stain (IF stain) and subcultured in 25 T MDCK cells (the passage 1, P1 virus). The P1 viruses were also checked by IF stain. Viruses were further amplified to P2 in MDCK cells and quantified by plaque assay for further analysis. The IF stain was done using D3 Ultra DFA Reagent Influenza A (Diagnostic Hybrids).

### Virus growth kinetics

To analyze the viral growth kinetics, A549 cells were seeded in 24-well plates in the concentration of 1.5 × 10^5^ cells/0.5 mL. After 24 h of culture, cells were infected by 0.5 mL of virus with 1 MOI (Fig. 4a) or 0.01 MOI (Fig. 4b and c) in virus culture medium containing 1.5 μg/mL trypsin. Viruses were collected after 0, 2, 4, 6, 8, 10, and 12 h post-infection for one-step growth curve and 0, 24, 48, and 72 h post-infection for multi-step growth curve. Collected virus samples were titrated by plaque assay.

### Plaque assay

MDCK cells were seeded in 12-well plates in the concentration of 5 × 10^5^ cells/mL. After 24 h of culture, cells were infected with 200 μL of ten-fold serially diluted viruses. Virus adsorption was carried out for 1 h at 35 °C and medium-agarose mixture were added to a total volume of 2 mL per well. Three days post-infection, cells were fixed with 10% formaldehyde and stained with 1% crystal violet.

### Quantitative RT-PCR assays for vRNA and mRNA

To evaluate vRNA and mRNA in virus-infected cells, A549 cells were seeded in 6-well plates in the concentration of 1 × 10^6^ cells/2 mL. After 24 h of culture, cells were infected with viruses at an MOI of 1. Total RNAs were extracted at 6 h post-infection and treated with DNase. Oligo-dT and influenza universal primer, uni-12, for mRNA and virus vRNA, respectively, were used in the RT reaction. For quantitative PCR, cDNAs were treated with RNase H, then amplified both influenza virus M gene (GACCRATCCTGTCACCTCTGAC and AGGGCATTYTGGACAAAKCGTCTA) and β-actin (CCAACCGCGAGAAGATGA and CCAGAGGCGTACAGGGATAG) with specific primers in the Roche Light Cycler 2.0. M gene probe (FAM-TGCAGTCCTCGCTCACTGGGCACG-BBQ) was used to detect viral mRNA and vRNA. β-actin (Universal ProbeLibrary probe #64, Roche) expression was also examined for each sample for normalization of gene expression between different samples.

### Virus infection and immunoblot analysis

A549 cells were infected with influenza viruses and whole-cell lysates were extracted with 1X lysis buffer at indicated hours post-infection. Lysates were centrifuged (13,000 rpm, 10 min, 4 °C) and the supernatants were collected for immunoblot assay. NP (ab128193, Abcam) and β-actin (A5411, Sigma) proteins were examined by virus-specific primary antibodies and HRP-labeled secondary antibodies (474–1802, KPL), as indicated.

### Ferret experiment

Adult ferrets were housed at the Peter Doherty Institute for Infection and Immunity Bioresources Facility. Male and female ferrets used in this study were 4–6 months old. Experiments were conducted with approval from the University of Melbourne Microbiology and Immunology Animal Ethics Committee, in accordance with the Australian National Health and Medical Research Council code of practice for the care and use of animals for scientific purposes. All ferrets were seronegative by haemagglutination inhibition assay for antibodies to currently circulating influenza viruses before use in experiments. The hemagglutination inhibition (HI) assay was conducted on day 0 before infection to ensure the ferrets have not been exposed to human seasonal influenza viruses. Influenza virus strains used in HI assay were A/Hong Kong/4801/2014 (H3N2), A/California/7/2009 (H1N1), B/Phuket/3073/2013-like, and B/Brisbane/60/2008-like. Sera from all these ferrets showed no detectable HI titers against tested strains, therefore being defined as seronegative.

Ferrets were infected intranasally with 2.5 × 10^5^ plaque-forming units (PFU) in 500 μL and monitored for 10 d.p.i. Four ferrets were housed by infection group. Nasal wash specimens were collected and stored. Nasal washes were collected daily until 6 d.p.i. Animals were weighed, visually inspected daily, and their temperature was measured using electronic identification chips with temperature monitoring. Nasal wash virus titers were expressed as 50% log10 tissue culture infectious doses (TCID50) [21, 22].

## Results

### NP gene evolution of H3N2 viruses

**Fig. 1:**
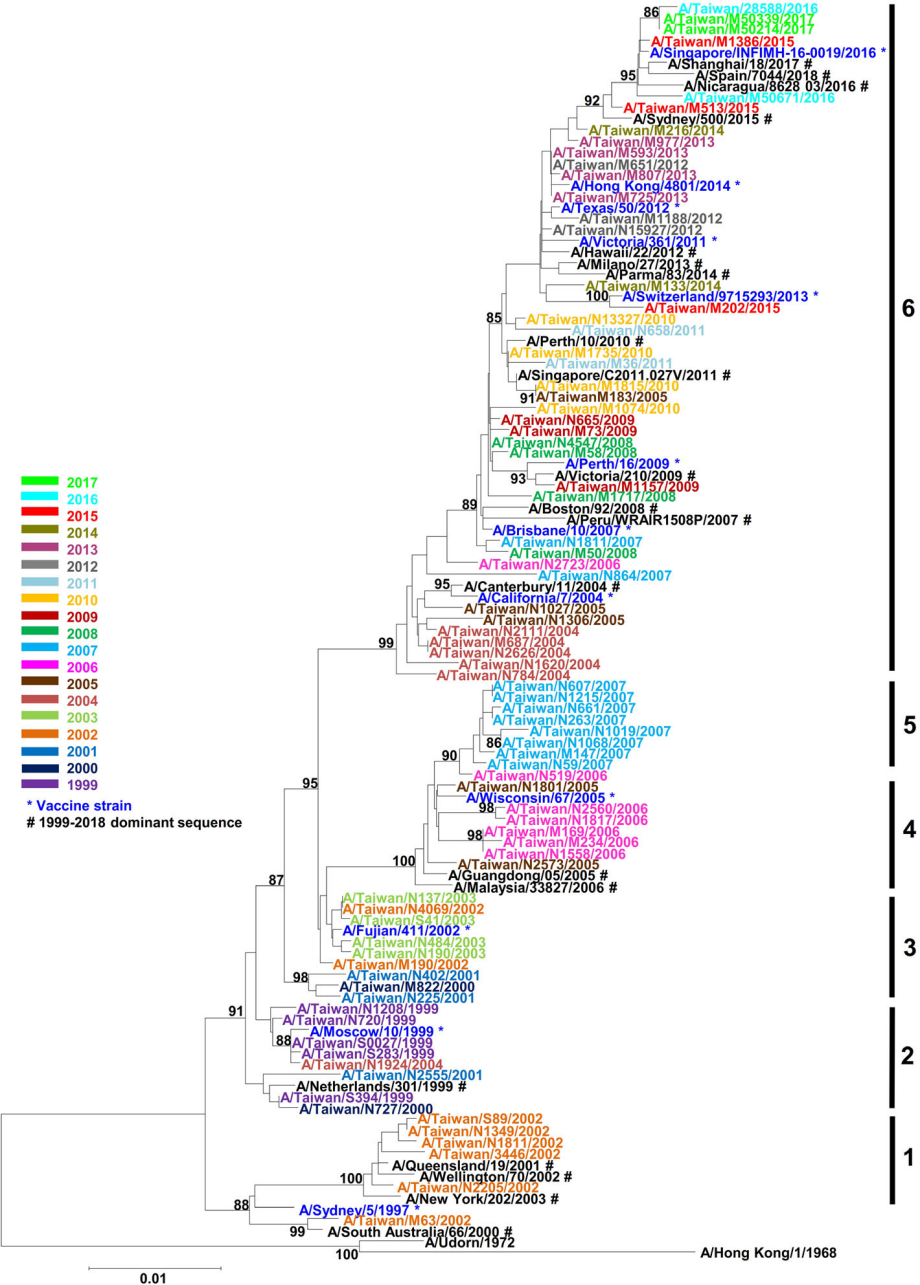
Phylogenetic analysis of NP of H3N2 viruses from 1999 to 2017. Phylogenetic analysis of influenza virus NP gene (nucleotide 16 to 1473 bases) was performed using MEGA 7. NP genes from 79 randomly selected Taiwan clinical isolates, 14 reference strains, as well as the most dominant sequences in each year (1999–2018), was separated into 1 to 6 clades. Bootstrap values over 85 were labeled

To understand the evolution pattern of the NP gene, influenza H3N2 clinical isolates from 1999 to 2017 from the Virology Laboratory of NCKUH were analyzed. The epidemiology curve showed that the H3N2 subtype continuously circulated in Taiwan and caused epidemics throughout the years (Additional file 1). Phylogenetic analysis of NP nucleotide sequences from 79 randomly chosen isolates from this period was showed in Fig. 1. The A/Hong Kong/68 H3N2 virus strain, isolated in the 1968 pandemic was used as the ancestral strain. Vaccine strains after 1997 were also included as reference strains. The NP gene did evolve over time, as shown by the progressively increasing distance from the ancestral strain. Moreover, this tree separated into various clades that we further termed clades 1–6 according to their amino acid substitutions. By phylogenetic analysis, we demonstrated that NP gene had accumulated many genetic changes and evolved into several clades with amino acid changes in protein (Additional file 2). Some substitutions were only observed during a specific time interval, for example, 27S, 103R, and 480E substitutions were identified in five of eight isolates in 2002; 77R and 425V substitutions before 2004. Some substitutions accumulated were more durable, for instance, 136I was first found in 2002, and conserved since 2003; 52H, 280A, and 312I changes were identified in 2004 and stayed conserved since 2007; 131S first appeared in 2005 and since then has been conserved until 2017 (Additional file 2).

To expand our findings and to overcome geography limitations focusing on a set of viruses from Taiwan, a total of 14,220 NP sequences of human H3N2 from the IRD were included to analyze the genetic variations of NP globally. The most dominant sequence in each year (the percentage showed in Table 1) was also included in the phylogenetic tree and showed similar time distribution among clades as Taiwan isolates. When comparing protein variations, the backbone of NP in majority showed significant change before and after 2004 (Table 1). The 27S-77R-103R-146A-425V-480E backbone was the most dominant circulating type from 2001 to 2003 (41.67, 75.13, and 52.81% in 2001, 2002, and 2003, respectively) while the 52H-98R-136I-146A-197V-280A-312I-406T composition was identified and became dominant since 2004. The 52H, 280A and 312I were stably conserved since 2007 and the backbone further acquired 131S within the same year. Since 2015, this backbone has had minor changes in sequence, with 197I and 472A gradually becoming dominant.

**Fig. 2:**
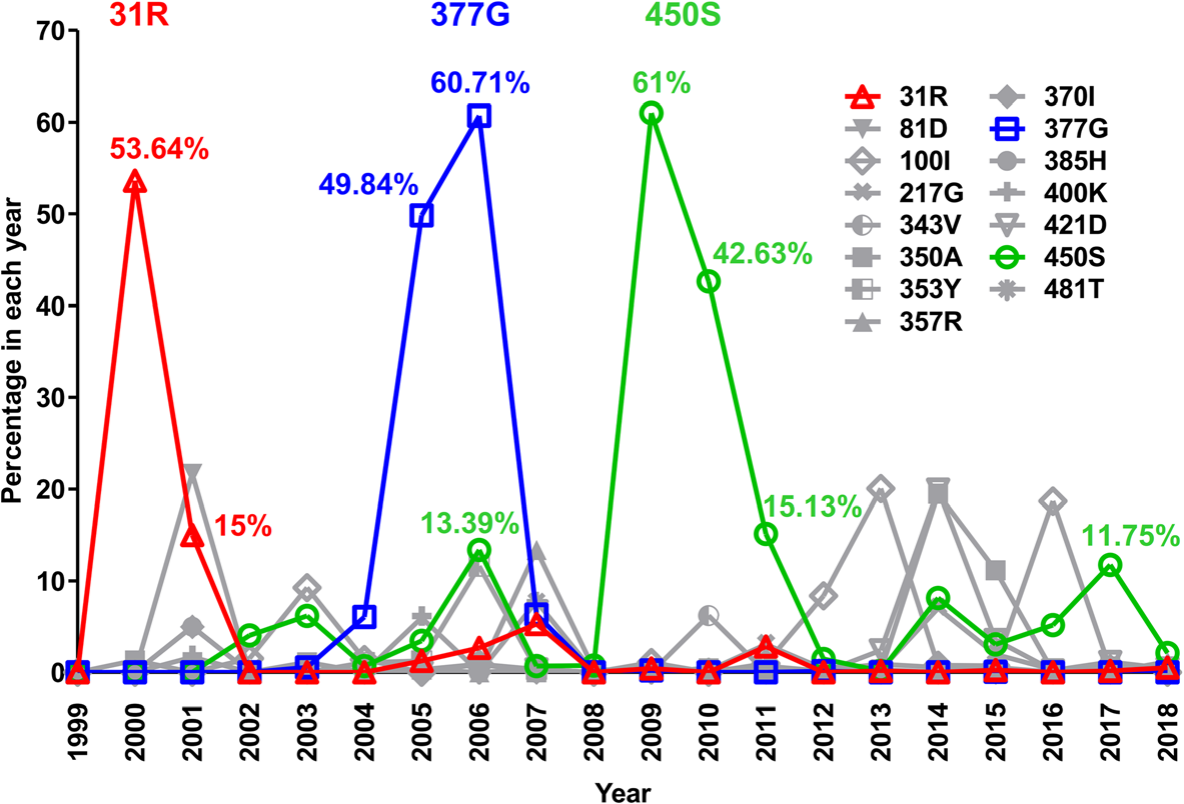
The 31R, 377G, and 450S substitutions showed higher ratio in specific years. Residues defined as sporadic mutations in Table 1 were further calculated their percentage in total global sequences in each year from 1999 to 2018. The percentage of more than 10% of residues 31R, 377G and 450S were labeled (31R in red, 377G in blue and 450S in green). To easily observed the curve of 31R, 377G and 450S, all other residues colored in gray but with different symbols

Other than those residues that composed the major sequence backbone, another 15 amino acid changes were observed. Although these changes, we defined them as sporadic substitutions, did not remain conserved in major backbone they appeared in some periods during evolution. Figure 2 showed the percentage of these changes from 1999 to 2018; among these, 31R, 377G, and 450S had been over 50% while each was dominant in a different year. The 31R was dominant in 2000 (53.64%) and was 15% in 2001 while showed low frequency in other years (from 0 to 5.28%) (Fig. 2). The 377G was dominant in 2005 (49.84%) as well as 2006 (60.71%) but showed low frequency in other years (from 0 to 6.34%). The 450S showed more than 10% in 2006 (13.39%), 2009, 2010, 2011 (61, 42.63 and 15.13%, respectively) and 2017 (11.75%). Therefore, substitutions at residues 31, 377 or 450 that appeared to have had a higher frequency in NP evolution history were further investigated. We hypothesized that during the virus evolution in human, these produced genetic variations in the internal genes of influenza virus and may play an important role in modulating certain virus properties.

### Effect of genetic variations of NP gene on polymerase activity

**Fig. 3:**
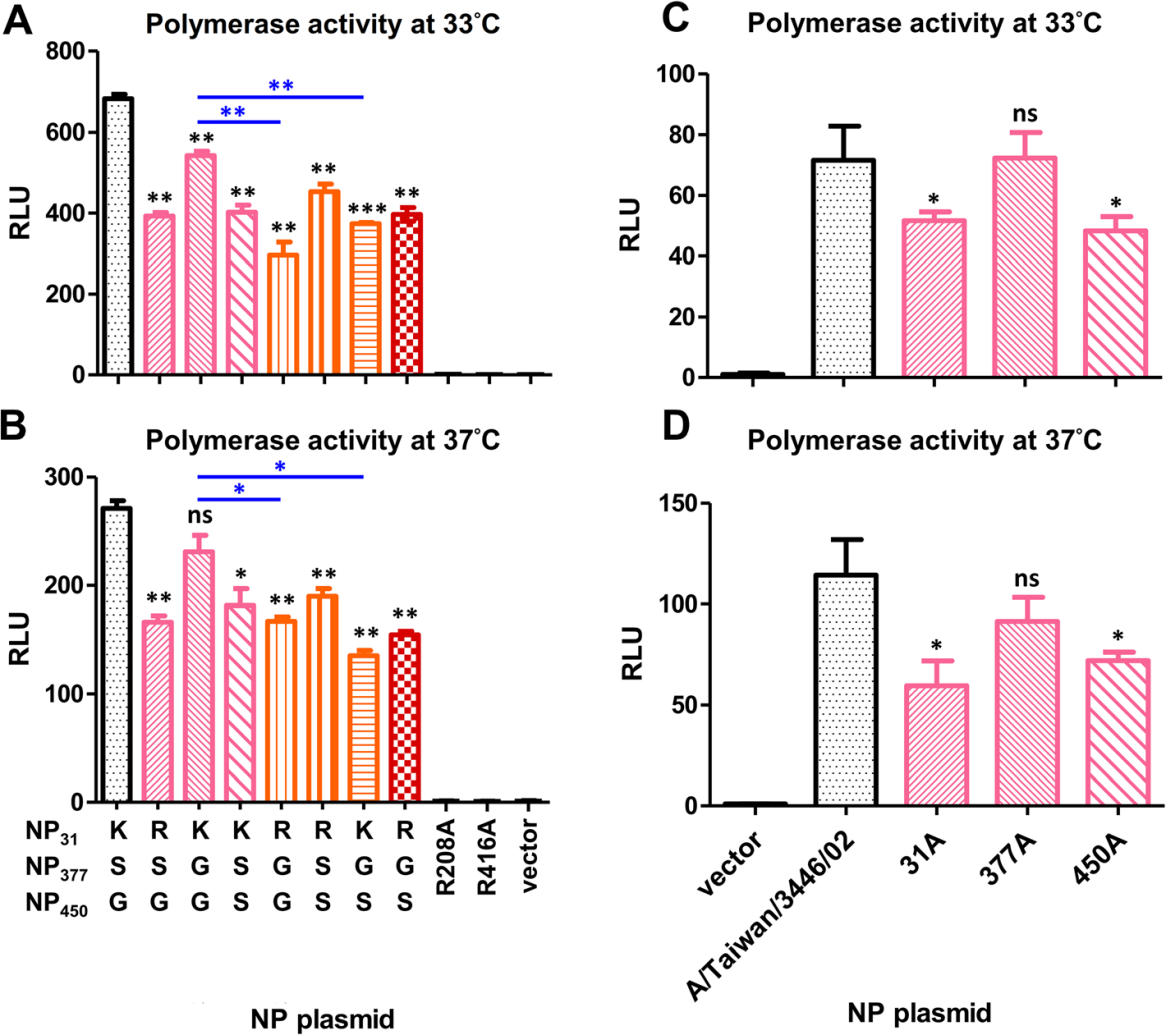
Variations on NP-31 and NP-450 residues affected polymerase activity. The mini-genome assay was carried out to analyze the polymerase activity with various point substitutions on NP at (**a**) 33 °C and (**b**) 37 °C. Polymerase activity with alanine point substitutions was also analyzed both at (**c**) 33 °C and (**d**) 37 °C. The X-axis showed the NP origins and the Y-axis showed the relative polymerase activity where firefly luciferase was normalized with renilla luciferase, internal control. The t-test value was calculated; *p < 0.05, **p < 0.01. (The black stars in (**a**) and (**b**) indicated comparison with NP_31_K-NP_377_S-NP_450_G, and in (**c**) and (**d**) indicated comparison with A/Taiwan/3446/02. Blue colored stars indicated a comparison between groups labeled with a line)

A mini-genome assay was commonly used to investigate viral RNA transcription/replication step, in which viral polymerases and NP play major roles. Alanine mutation at residues 208 and 416 served as negative controls according to previous studies [5, 23]. To examine whether genetic variations at these three positions altered polymerase activity, A/Taiwan/3446/02 NP (31 K-377S-450S) was used as a backbone to generate mutations for analysis. Polymerase genes from A/Taiwan/3446/02 strain were used as backbone for this assay. By site-directed mutagenesis, single, double and triple mutations at residues 31, 377 and 450 of NP were generated. The relative polymerase activity pattern between different NPs were similar both at 33 °C and 37 °C (Fig. 3a and b). Amino acid change at residue 450 (from G to S) statistically decreased polymerase activity as shown when comparing NP_31_K-NP_377_S-NP_450_G (KSG) with NP_31_K-NP_377_S-NP_450_S (KSS) and NP_31_K-NP_377_G-NP_450_G (KGG) with NP_31_K-NP_377_G-NP_450_S (KGS). The single substitution at residue 377 (when comparing KSS with KGS, and RSG with RGG at both temperatures; KSG with KGG at 37 °C and RSS with RGS at 33 °C) did not result in a significant change of activity. Although it showed no significant difference when comparing KSS with RSS, polymerase activity between KSG and RSG as well as between KGG and RGG showed statistical differences that demonstrated the effect of substitution at residue 31 (from K to R). Thus, we concluded that residues 31 and 450 modulated the polymerase activity and NP with 450G had a higher activity while 31R downregulated polymerase activity. To further investigate the role of residues 31, 377 and 450 of NP on polymerase activity, alanine substitutions were analyzed. Both 31A and 450A, but not 377A, statistically decreased polymerase activity when compared with A/Taiwan/3446/02 NP both at 33 °C and 37 °C (Fig. 3c and d). Thus, we suggest alanine substitution at 31 and 450 of NP dramatically decreased the RdRP activity. Because either mutation in residues 31 or 450 would significantly decrease the polymerase activity, except residue 377, both residues 31 and 450 were critical in regulating the polymerase activity.

### Effect of substitutions on virus replication

**Fig. 4:**
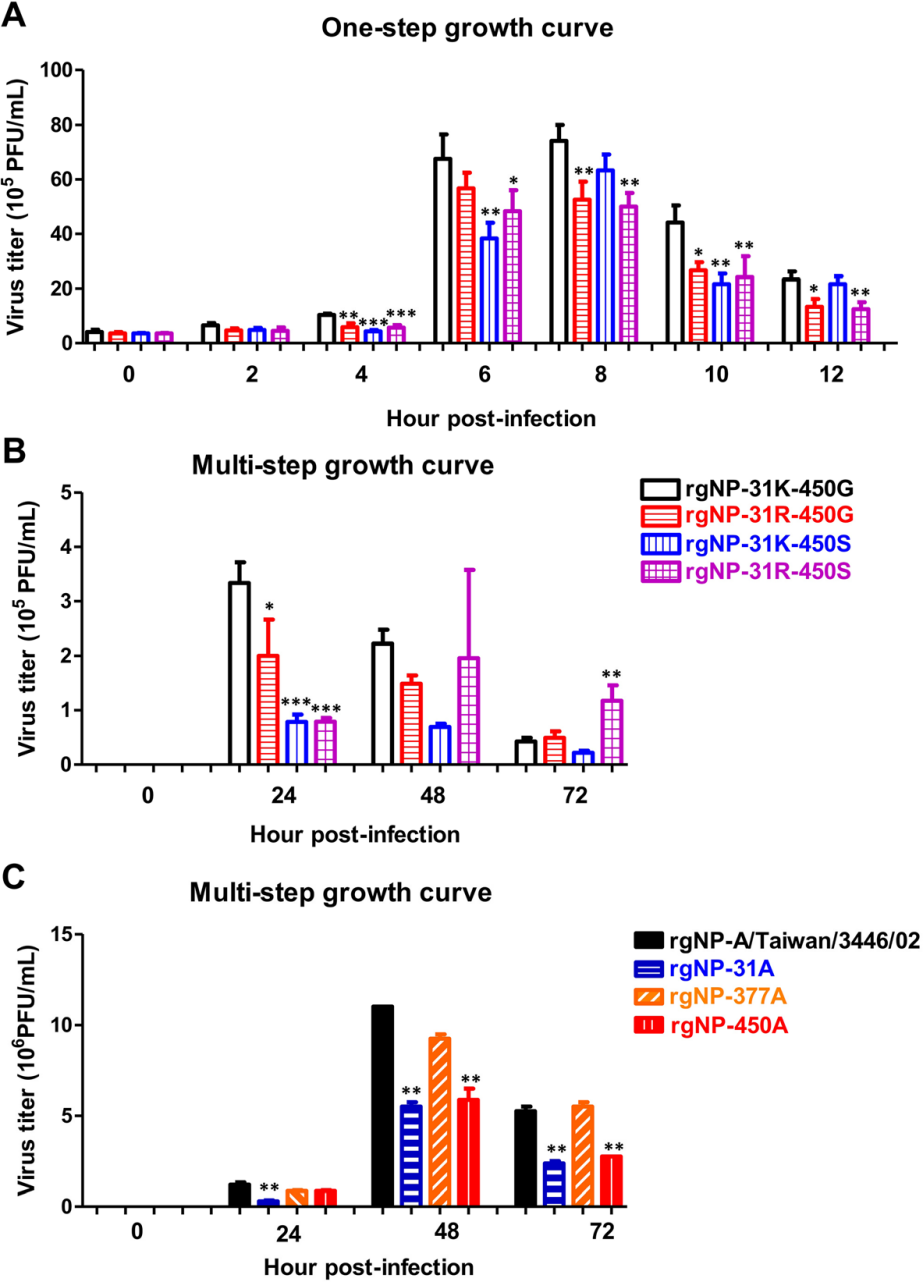
Variations on NP-31 and NP-450 residues affected viral growth kinetics. A549 cells were infected with reverse genetics with different amino acid composition as indicated at (**a**) MOI of 1 and (**b**)(**c**) MOI of 0.01 at 37 °C. At the selected time points, viruses were harvested and examined by plaque assay in MDCK cells. The one-way ANOVA with Tukey post-test was performed; *p < 0.05, **p < 0.01, ***p < 0.001

After examining the effect of these residues by mini genome assay, we next aimed to investigate whether they affected virus replication. To achieve this goal, a reverse genetics system was used to produce viruses with specific mutations on NP. In this system, the A/Taiwan/3446/02 strain served as genetic backbone that provided the other seven gene segments to rule out other gene effects on virus replication. To examine the viral growth properties of viruses with variant residues at 31 and 450 of NP, one-step replication and multi-step replication cycles in A549 cells were examined at a MOI of 1 and a MOI of 0.01, respectively (Fig. 4a and b). The results indicated that viruses with 31K-450G had higher viral titers than 31R-450G, 31K-450S, and 31R-450S from 4 h post-infection in one-replication cycle as well as 24 h post-infection in multi-step growth curve. These findings demonstrated the substitutions found in the NP evolution changed viral growth properties that may act via regulation of the polymerase activity. Reverse genetics viruses with alanine mutation at residues 31, 377 and 450 were also analyzed. We observed that virus with 31A or 450A statistically decreased viral growth, whereas 377A had similar growth kinetics as rg-A/Taiwan/3446/02 virus (Fig. 4c).

### Effect of substitutions on viral replication, transcription and translation

**Fig. 5:**
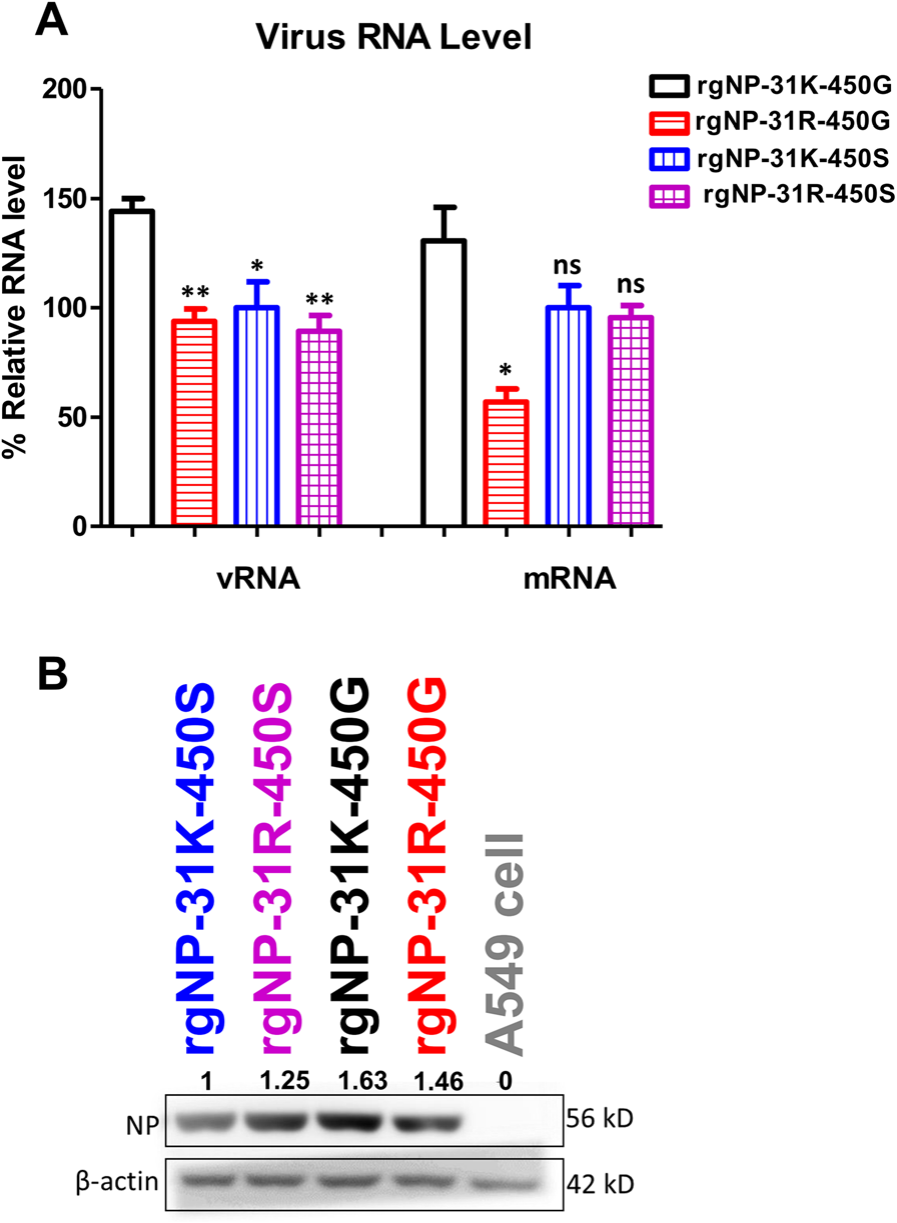
Two substitutions affected viral replication, transcription and translation. **a** Virus RNA level in A549 cells at 6 h post-infection was investigated. Levels of M genes were estimated by qRT-PCR and normalized with the β-actin genes of cells. Negative-sense viral vRNA and positive sense viral mRNA were compared. The one-way ANOVA with Tukey post-test was performed; *p < 0.05, **p < 0.01. **b** Virus NP protein levels in A549 cells at 9 h post-infection were examined. The expression level of NP and β-actin were examined by Western blot and analyzed by Quantity One software

NP has a major role in RNP complex and can regulate polymerase activity which may change viral growth properties. To dissect the hypothesis that polymerase activity alteration can affect viral growth titer, we further investigated viral transcription, replication, and translation. A549 cells were infected with mutant viruses at a MOI of 1, and the viral RNAs were extracted. The level of M gene was normalized with the β-actin gene that was quantitated by qRT-PCR. Compared with the RNA level of NP-31K-450G, the 31R-450G and 31K-450S as well as 31R-450S substitutions decreased vRNA and mRNA levels (Fig. 5a). Regarding translation, virus-infected cells were lysed and immunoblot analysis was performed to measure the amount of NP protein present. Similar patterns were observed whereby the virus rgNP-31K-450G expressed the highest protein levels, but viruses with substitutions showed slightly decreased protein levels (Fig. 5b).

### The virus of NP-450G had prolonged shedding in nasal washes of ferrets

**Fig. 6:**
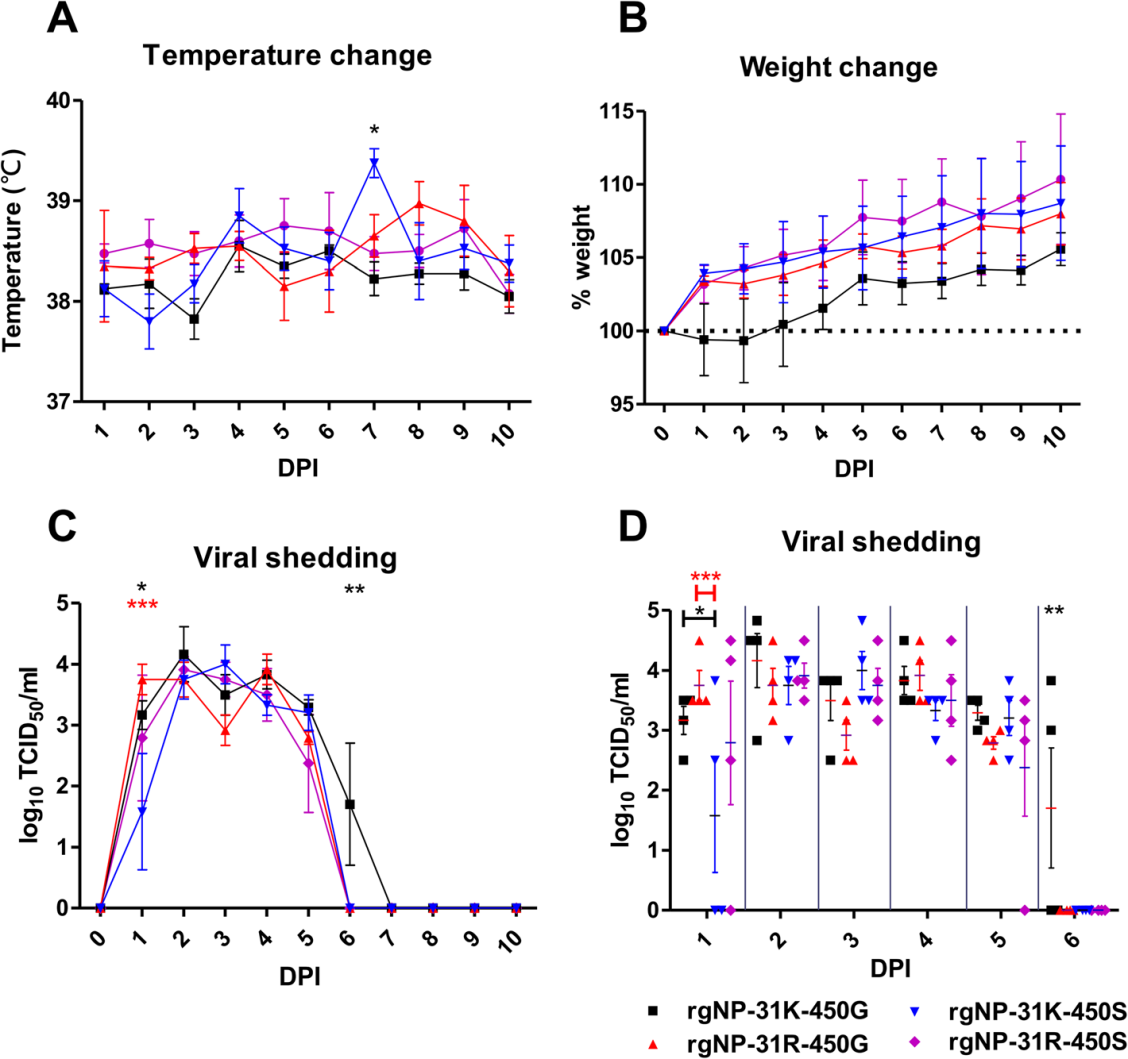
Weight, temperature change and viral shedding in nasal wash of ferrets. Ferrets were infected intranasally with 2.5 × 10^5^ plaque-forming units (PFU) in 500 μL of viruses with NP substitutions. **a** Temperature and (**b**) weight changes were monitored. Nasal wash specimens were collected and expressed as 50% log10 tissue culture infectious doses (TCID50). In (**c**) viral shedding curve and (**d**) viral shedding dot plot, viruses with different substitutions were separated by colors and shapes; each dot represents the viral titer in nasal wash of each ferret. Two-way ANOVA, multiple comparisons with Bonferroni post-hoc test was performed; *p < 0.05, **p < 0.01, ***p < 0.001

We next examined the effect of the substitutions in vivo. Ferrets were challenged with reverse genetics viruses with different amino acid composition in residue 31 and 450 of NP intranasally on day 0. The temperature of the ferrets showed no significant changes in any of the groups tested following challenge, and all ferrets showed weight gains post challenge but ferrets from the rgNP-31K-450G group had a slower rise in body weight gain for the first 4 days after virus challenge, whereas ferrets from the other groups all had similar weight gains (Fig. 6a and b). Viral loads in nasal washes were assessed daily following challenge (Fig. 6c and d), with ferrets from the rgNP-31K-450G and rgNP-31R-450G groups shedding virus from day one post-challenge and achieving viral titers that were statistically higher than rgNP-31K-450S and rgNP-31R-450S groups. Noticeably, the viral titers of two ferrets in rgNP-31K-450S and one in rgNP-31R-450S were undetectable at day 1-post challenge. In addition, ferrets from the rgNP-31K-450G group continued to shed virus at low levels at day 6 post-challenge, while ferrets in all other groups had ceased shedding virus at this timepoint.

## Discussion

Through the analysis of NP gene evolution in Taiwan and globally, we observed that the protein sequence backbone had a great change around 2004 and since then the NP still continuously attained new changes year by year. Moreover, we identified fifteen sporadic substitutions and those who had higher frequencies (31R, 377G and 450S) were further analyzed by in vitro and in vivo assays. The 31R and 450S were confirmed to reduce viral replication while the dominant circulating residues (31 K and 450G) were beneficial to viral replication properties. In this study, we evaluated NP substitutions in evolution and demonstrated amino acid changes in 31 and 450 of NP could modulate virus replication.

To understand the basic functions of NP, many studies have paid attention to residues of the conserved region and defined several domains on NP, such as RNA binding, PB2 binding, oligomerization (NP-NP interaction) and NLS. However, crucial residues may exist at sites that have yet to be identified. Via alanine mutation and reverse genetics system, 74 conserved residues among influenza viruses were analyzed and their impact on virus replication or RNA incorporation were identified [10]. For example, R208A and R416A, which served as negative control in the mini-genome assay were shown to have very low polymerase activity and failed to rescue as reverse genetics viruses. There have been several evolutionary analyses focused on the NP gene segment in recent years [24, 25] and by phylogenetic analysis, separate clades from human, avian or swine origins can be identified. Some positive selection sites on NP were found, one of which was residue 450 [16]. The 31R substitutions identified here was also observed by the Centers for Disease Control of Taiwan, however, the effects of substitution have not been investigated until now [19]. Owing to the multiple roles of NP, there is no specific assay for analyzing the functional variability or the effect of mutations in NP. The mini-genome assay is used widely to investigate the impact on virus replication, and the major role of NP in virus infection and replication. Therefore, we applied this assay to screen the evolution of genetic variations and further demonstrated their effects on virus replication by examining these changes in viruses generated by reverse genetics. In this study, genetic variations of NP observed in evolution were further analyzed and we demonstrated that NP-31 and NP-450 may have important effects on virus replication.

Specifically, we found that NP with 450G significantly enhanced viral growth in vitro, also prolonged virus shedding in vivo slightly. In our ferret study, neither the body temperature change nor the weight loss showed any comparative differences. The rgNP-31K-450G virus-infected ferrets did however show slower gain in weight post-challenge and shed virus for a day longer compared to the rgNP-31K-450S virus challenged ferrets. To investigate whether this enhancement of viral properties correlated with larger influenza outbreaks or disease severity, we looked at the human seasonal influenza (H3N2) epidemiology for Taiwan. Notably, viruses with NP-450G was the dominant circulating strain since 1968, whereas in the 2006–2007 influenza virus season, NP-450S appeared temporarily in Taiwan (Additional file 2). The excess morbidity of H3N2 was reported as being low in 2006–2007, with only 0.98 per 100,000 population, compared to 2003–2005 with an average range value of 3.06–6.01 per 100,000 population [26]. Thus, the effect of the evolutionary variations that we investigated in vitro can also reflect on the ferrets in vivo study, which can also in turn reflect on human epidemiology. Here, we tried to correlate with epidemiology reports, whereby NP-450S reduce morbidity in the 2006–2007 influenza virus season, however, there are still many concerns and/or parameters that could not exactly be filtered out, therefore, further study is required.

Other studies have shown the influence of NP on virulence and pathogenicity. Amino acid differences at residues 50 and 98 on NP have shown to result in high intracerebral pathogenicity in chicken by a duck origin, low pathogenic H7N1 influenza virus [27]. Positions 105 and 184 of NP contribute to virus replication and pathogenicity of avian H5N1 influenza virus [28, 29]. The substitution NP-D375N of 2009 pandemic H1N1 virus resulted in better adaption to mice [30]. These studies indicated that genetic variations on NP affect not only the pathogenicity but also host adaptation. In other studies, the changes in pathogenicity by NP-D101G and NP-N109T substitutions resulted in the modulation of polymerase activity [31, 32]. Thus, in addition to NP residues identified to date, more work is required to better understand the role of NP in human influenza evolution and fitness. Avian influenza infection is a great threat to humans and it is important to understand the mechanism of host adaptation. The E627K substitution of PB2 is a well-known case where a single mutation contributes to enhancing replication of avian influenza in mammals [33]. The NP-N319K mutation affects the interaction of NP with host factor importin-α and leads to an increase in virus replication [34]. Although there were a few adaptive mutations identified to date, further investigation of NP may reveal more novel aspects that NP plays in host adaptation since NP has multiple roles in virus replication and interacts with various host factors.

Increasingly more studies have focused on the viral whole genome sequence analysis and have tried to understand the complicated mechanisms of influenza virus evolution and clinical outcome. For instance, the severe H3N2 epidemic of the 2003–2004 flu season led to high mortality, especially in children [16]. The study observed many more amino acid substitutions not only on the HA but also in the other seven gene segments from the dominant strain of previous season. Analysis of the human H3N2 whole genome sequences from databases revealed that there are evolutions in codon usage and frequent reassortment events [35–37]. A Previous study showed that reassortment occurred whereby A/Sydney/5/97 converted to A/Fujian/411/02 strain, and A/Fujian/411/02 converted to A/California/7/04 strain [37]. In our phylogenetic data, clade 1, where sequences were similar to A/Sydney/5/97, there was a clade separated from the other clades 2–6. In clade 6, progressive drift from A/California/7/04 was observed, in contrast to clades 2 to 5. This demonstrates that the NP genetic variations observed in our and other studies come from not only the RNA genome mutations but may also involve the reassortment mechanism.

In this study, substitutions of NP analyzed by mini-genome assay demonstrated NP-31 and NP-450 are important determinants in NP activity. Though NP-377, which we also found in the evolutionary history of influenza, did not affect polymerase activity significantly, a recent study had demonstrated that NP-377 is a phosphorylation site in the H1N1 WSN strain. Amino acids sequence alignment of WSN and our A/Taiwan/3446/02 strain showed that NP had an 89.3% identity and NP-377 was conserved. A previous study showed that mutated phosphorylated residues reduced viral titer [38], and the same effect was observed in our study whereby 377G slightly decreased the polymerase activity. Thus, the post-translational modification may also have some functions and participate in NP’s evolution.

Epistasis, which is a term used in genetics and evolution, is defined as some mutations being tolerated only after the occurrence of others that can have either positive or negative effects [39]. In evolution, epistasis may play a key role in immune escape and drug resistance [40]. Epistasis was found in the NA protein of influenza H1N1 virus and contributed to the emergence of resistant strains [41]. Gong and Bloom predicted the evolution trajectory of H3N2 viruses from Aichi/1968 to Brisbane/2007 strain by computational analysis and found that the epistatically constrained variations were destabilizing and had required another mutation to stabilize the NP [42]. They demonstrated three single mutations (L259S, R384G, and V280A) which decreased polymerase activity, but the gain of other mutations before these mutations had occurred can rescue their deleterious effect. Some of the residues of NP backgrounds before and after 2004 that we had identified by the global evolution were included in their predicted trajectory and the V280A was demonstrated to be under the control of epistasis. In this study, we focused on those sporadic substitutions which were not analyzed by previous studies and showed that 31R and 450S decreased polymerase activity and they may need other mutations to stabilize and to support conservation through evolution.

## Conclusions

Instead of analyzing surface proteins of HA and NA of influenza A(H3N2) viruses, we analyzed the NP gene of human influenza A H3N2 viruses that have circulated from 1999 to 2018. NP-31 and NP-450 were identified as possible sites that affect polymerase activity and also had an effect on viral replication, transcription, translation, growth kinetics, and in a ferret infection study. In conclusion, we demonstrated that two NP protein substitutions acquired from evolution appear to have an impact on some influenza viral properties. Hence, we suggest that the evolutional genetic variations of NP should be continuously monitored, and their effects determined.

## Supplementary information

**Additional file 1.** Epidemiology of H3N2 viruses from 1999 to 2017 in Taiwan. Isolated cases of influenza A H3N2 viruses from Virology Laboratory of National Cheng Kung University Hospital.

**Additional file 2.** Amino acid substitutions in NP protein of H3N2 viruses. NP substitutions of 79 randomly chosen Taiwan H3N2 isolates from 1999 to 2017, WHO recommended vaccine strains and reference strains were compared.

## Data Availability

All data generated or analyzed during this study are included in this published article [and its supplementary information files].

## Abbreviations

IF stain: immunofluorescence stain
IRD: Influenza Research Database
NCKUH: National Cheng Kung University Hospital
NLS: nuclear localization signal domain
NP: nucleoprotein
P/S: Penicillin/Streptomycin
PFU: plaque-forming units
RNP: ribonucleoprotein
TCID50: 50% log10 tissue culture infectious doses
WHO: World Health Organization
