# Supplementary figures and images for: Genetic variations on 31 and 450 residues of influenza A nucleoprotein affect viral replication and translation
Source: J Biomed Sci. 2020 Jan 6;27:17. doi: 10.1186/s12929-019-0612-z (PMC6943894; doi:10.1186/s12929-019-0612-z)

# Epidemiology of H3N2 viruses

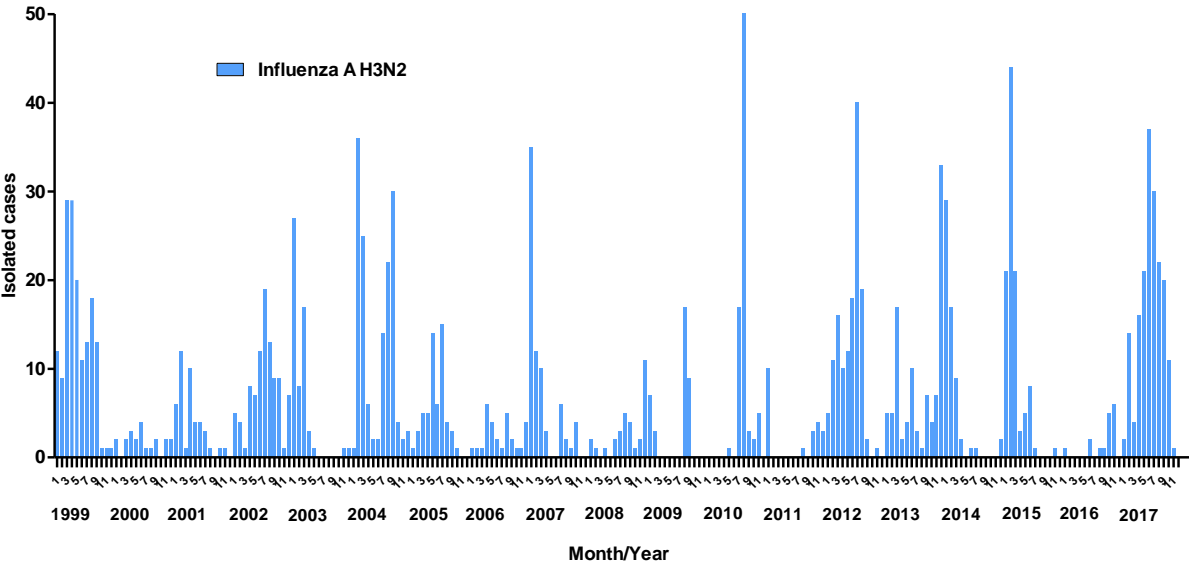

Supplement: Supplementary file 1 — Additional file 1. Epidemiology of H3N2 viruses from 1999 to 2017 in Taiwan. Isolated cases of influenza A H3N2 viruses from Virology Laboratory of National Cheng Kung University Hospital. [file 12929_2019_612_MOESM1_ESM.pdf]
