# Supplementary material for: Genetic variations on 31 and 450 residues of influenza A nucleoprotein affect viral replication and translation
Source: J Biomed Sci. 2020 Jan 6;27:17. doi: 10.1186/s12929-019-0612-z (PMC6943894; doi:10.1186/s12929-019-0612-z)
Supplement: Supplementary file 2 — Additional file 2. Amino acid substitutions in NP protein of H3N2 viruses. NP substitutions of 79 randomly chosen Taiwan H3N2 isolates from 1999 to 2017, WHO recommended vaccine strains and reference strains were compared. [file 12929_2019_612_MOESM2_ESM.pdf]

Additional file 2. Amino acid substitutions in NP protein of H3N2 viruses

	NP amino acid position																																																							
	18	27	31	50	52	65	77	81	85	95	98	101	103	114	127	128	131	136	146	186	197	217	239	280	286	287	301	312	334	343	350	353	354	360	363	371	373	375	377	384	396	400	406	411	417	421	423	425	430	450	452	456	459	472	480	481	482
Hong Kong/1/68	E	A	K	S	Y	R	K	E	A	P	K	D	K	E	D	D	A	M	T	V	I	S	M	V	A	S	I	V	N	V	T	S	P	T	V	M	A	E	S	R	T	R	I	A	N	D	P	I	T	G	K	M	Q	A	D	M	S
Udorn/72						R																G								L							T										S					V	R	T			
Sydney/5/97	D					K	R				R		R		E					I	V		V		S				H	L							N	G		G				T		E	S	V				V	R	T			
Moscow/10/99	D			N		K					R				E				A	I	V		V		S				H	L							N	G		G				T		E	S	V				V	R	T			
Fujian/411/02	D					K					R				E			I	A	I	V		V		S				H	L							N	G		G			T	T		E	S					V	R	T			
California/7/04	D				H	K					R				E			I	A	I	V		V	A	S			I	H	L	K						N	G		G			T	T		E	S					V	R				
Wisconsin/67/05	D					K					R				E			I	A	I	V		V		S				H	L							N	G	G	G			T	T		E	S					V	R	T			
Brisbane/10/07	D				H	K					R				E		S	I	A	I	V		V	A	S			I	H	L							N	G		G			T	T		E	S					V	R	T			
Perth/16/09	D				H	K					R				E		S	I	A	I	V		V	A	S			I	H	L							N	G		G			T	T		E	S			S		V	R	T			
Victoria/361/11	D				H	K					R	G		D	E		S	I	A	I	V		V	A	S			I	H	L							N	G		G			T	T		E	S			S		V	R	T			
Texas/50/12	D				H	K					R				E		S	I	A	I	V		V	A	S			I	H	L							N	G					T	T		E	S			S		V	R	T			
Switzerland/9715293/13	D				H	K					R				E		S	I	A	I	V	G	V	A	S			I	H	L							N	G		G			T	T		E	S			S		V	R	T			
Hong Kong/4801/14	D				H	K					R				E		S	I	A	I	V		V	A	S			I	H	L							N	G		G			T	T		E	S			S		V	R	T			
A/Singapore/INFIMH-16-0019/2016	D				H	K					R				E		S	I	A	I	V		V	A	S			I	H	L							N	G		G			T	T		E	S					V	R				
Taiwan/S0027/99	D					K					R				E				A	I	V		V		S				H	L							N	G		G				T		E	S	V				V	R	T			
Taiwan/S283/99	D					K					R				E				A	I	V		V		S				H	L							N	G		G				T		E	S	V				V	R	T			
Taiwan/S394/99	D					K	R				R				E					I	V		V		S				H	L							N	G		G				T		E	S	V				V	R	T			
Taiwan/N720/99	D					K					R				E				A	I	V		V		S				H	L							N	G		G				T		E	S	V				V	R	T			
Taiwan/N1208/99	D					K					R				E				A	I	V		V		S				H	L							N	G		G				T		E	S	V				V	R	T			
Taiwan/N727/00	D					K	R				R				E	G				I	V		V		S				H	L							N	G		G				T		E	S	V				V	R	T			
Taiwan/M822/00	D					K					R				E				A	I	V		V		S				H	L							N	G		G			T	T		E	S					V	R	T			
Taiwan/N402/01	D					K			T		R				E				A	I	V		V		S				H	L							N	G		G			T	T		E	S					V	R	T			
Taiwan/N225/01	D					K					R				E				A	I	V		V		S				H	L				I			N	G		G	I		T	T		E	S					V	R	T			
Taiwan/N2555/01	D					K	R	D			R				E			I		I	V		V		S				H	L			Q				N	G		G				T		E	S	V				V	R	T			
Taiwan/S89/02	D	S				K	R						R		E				A	I			V		S				H	L							N	G		G				T		E	S	V				V	R	T	E		
Taiwan/N1349/02	D	S				K	R						R		E				A	I			V		S				H	L							N	G		G				T		E	S	V				V	R	T	E		
Taiwan/N2205/02	D	S				K	R						R		E				A	I			V		S				H	L							N	G		G				T		E	S	V				V	R	T	E		
Taiwan/N1811/02	D	S				K	R						R		E				A	I			V		S				H	L							N	G		G				T		E	S	V				V	R	T	E		
Taiwan/3446/02	D	S				K	R						R		E				A	I			V		S				H	L							N	G		G				T		E	S	V		S		V	R	T	E		
Taiwan/N4069/02	D					K					R				E			I	A	I	V		V		S				H	L							N	G		G			T	T		E	S					V	R	T			
Taiwan/M190/02	D					K				S	R				E			I	A	I	V		V		S				H	L							N	G		G			T	T		E	S					V	R	T			
Taiwan/M63/02	D		R			K	R				R				E					I	V		V		S				H	L							N	G		G				T	D	E	S	V				V	R	T	E		

Additional file 2 (Continued). Amino acid substitutions in NP protein of H3N2 viruses

	NP amino acid position																																																							
	18	27	31	50	52	65	77	81	85	95	98	101	103	114	127	128	131	136	146	186	197	217	239	280	286	287	301	312	334	343	350	353	354	360	363	371	373	375	377	384	396	400	406	411	417	421	423	425	430	450	452	456	459	472	480	481	482
Hong Kong/1/68	E	A	K	S	Y	R	K	E	A	P	K	D	K	E	D	D	A	M	T	V	I	S	M	V	A	S	I	V	N	V	T	S	P	T	V	M	A	E	S	R	T	R	I	A	N	D	P	I	T	G	K	M	Q	A	D	M	S
Taiwan/N484/03	D					K					R				E			I	A	I	V		V		S				H	L							N	G		G			T	T		E	S					V	R	T			
Taiwan/N190/03	D					K					R				E			I	A	I	V		V		S	N			H	L							N	G		G			T	T		E	S					V	R	T			
Taiwan/N137/03	D					K					R				E			I	A	I	V		V		S				H	L							N	G		G			T	T		E	S					V	R	T			
Taiwan/S41/03	D					K					R				E			I	A	I	V		V		S				H	L							N	G		G			T	T		E	S					V	R	T			
Taiwan/N1924/04	D					K					R				E				A	I	V		V		S				H	L							N	G		G				T		E	S	V				V	R	T			
Taiwan/N1620/04	D			T		K					R				E			I	A	I	V		V	A	S			I	H	L							N	G		G			T	T		E	S					V	R	T			
Taiwan/M687/04	D				H	K					R				E			I	A	I	V		V	A	S			I	H	L							N	G		G			T	T		E	S					V	R	T			
Taiwan/N2626/04	D				H	K					R				E			I	A	I	V		V	A	S			I	H	L							N	G		G			T	T		E	S					V	R	T			
Taiwan/N2111/04	D				H	K					R				E			I	A	I	V		V	A	S	N		I	H	L							N	G		G			T	T		E	S					V	R	T			
Taiwan/N784/04	D					K					R				E			I	A	I	V		V	A	S			I	H	L							N	G		G			T	T		E	S					V	R	T			
Taiwan/N1027/05	D				H	K					R				E			I	A	I	V		V	A	S			I	H	L							N	G		G			T	T		E	S					V	R	T			
Taiwan/N1306/05	D				H	K					R				E			I	A	I	V		V	A	S			I	H	L							N	G		G			T	T		E	S					V	R	T		R	
Taiwan/M183/05	D				H	K			S		R				E		S	I	A	I	V		V	A	S			I	H	L							N	G		G			T	T		E	S					V	R	T			
Taiwan/N1801/05	D					K					R				E			I	A	I	V		V		S				H	L							N	G	G	G			T	T		E	S					V	R	T			
Taiwan/N2573/05	D					K					R				E			I	A	I	V		V		S				H	L							N	G		G			T	T		E	S					V	R	T			
Taiwan/N1558/06	D					K					R				E			I	A	I	V		V		S				H	L						I	N	G	G	G			T	T		E	S			S		V	R	T			
Taiwan/M169/06	D					K					R				E			I	A	I	V		V		S				H	L						I	N	G	G	G			T	T		E	S			S		V	R	T			
Taiwan/M234/06	D					K					R				E			I	A	I	V		V		S				H	L						I	N	G	G	G			T	T		E	S			S		V	R	T			
Taiwan/N1817/06	D					K					R				E			I	A	I	V		V		S				H	L		Y					N	G	G	G			T	T		E	S				R	V	R	T			
Taiwan/N519/06	D		R			K					R				E			I	A	I	V		V		S				H	L							N	G	G	G			T	T		E	S					V	R	T			N
Taiwan/N2560/06	D					K					R				E			I	A	I	V		V		S				H	L		Y					N	G	G	G			T	T		E	S				R	V	R	T			
Taiwan/N2723/06	D				H	K					R				E			I	A	I			V	A	S			I	H	L							N	G		G			T	T		E	S					V	R	T			
Taiwan/N263/07	D		R			K					R				E			I	A	I	V		V		S		V		H	L							N	G	G	G			T	T		E	S					V	R	T			
Taiwan/N661/07	D		R			K					R				E			I	A	I	V		V		S		V		H	L							N	G	G				T	T		E	S					V	R	T			
Taiwan/N1215/07	D		R			K					R				E			I	A	I	V		V		S				H	L							N	G	G	G			T	T		E	S					V	R	T			
Taiwan/M147/07	D		R			K					R				E			I	A	I	V		V		S				H	L							N	G	G	G			T	T		E	S					V	R	T			
Taiwan/N1068/07	D		R			K					R				E			I	A	I	V		V		S				H	L							N	G	G	G			T	T		E	S					V	R	T			
Taiwan/N59/07	D		R			K					R				E			I	A	I	V		V		S				H	L							N	G	G	G			T	T		E	S		I			V	R	T			
Taiwan/N607/07	D		R			K					R				E			I	A	I	V		V		S				H	L							N	G	G	G			T	T		E	S					V	R	T			
Taiwan/N1019/07	D		R			K					R				E			I	A	I	V		V		S				H	L					G		N	G	G	G			T	T		E	S					V	R	T			

Additional file 2 (Continued). Amino acid substitutions in NP protein of H3N2 viruses

	NP amino acid position																																																							
	18	27	31	50	52	65	77	81	85	95	98	101	103	114	127	128	131	136	146	186	197	217	239	280	286	287	301	312	334	343	350	353	354	360	363	371	373	375	377	384	396	400	406	411	417	421	423	425	430	450	452	456	459	472	480	481	482
Hong Kong/1/68	E	A	K	S	Y	R	K	E	A	P	K	D	K	E	D	D	A	M	T	V	I	S	M	V	A	S	I	V	N	V	T	S	P	T	V	M	A	E	S	R	T	R	I	A	N	D	P	I	T	G	K	M	Q	A	D	M	S
Taiwan/N864/07	D				H	K					R				E			I	A	I	V		V	A	S			I	H	L							N	G		G			T	T		E	S					V	R				
Taiwan/N1811/07	D				H	K					R				E		S	I	A	I	V		V	A	S			I	H	L							N	G		G			T	T		E	S					V	R	T		T	
Taiwan/M50/08	D				H	K					R				E		S	I	A	I	V		V	A	S			I	H	L							N	G		G			T	T		E	S					V	R	T		T	
Taiwan/M58/08	D				H	K					R				E		S	I	A	I	V		V	A	S			I	H	L							N	G		G			T	T		E	S					V	R	T			
Taiwan/N4547/08	D				H	K					R				E		S	I	A	I	V		V	A	S			I	H	L							N	G		G			T	T		E	S					V	R	T			
Taiwan/M1717/08	D				H	K					R				E		S	I	A	I	V		V	A	S			I	H	L							N	G		G			T	T		E	S					V	R	T			
Taiwan/M73/09	D				H	K					R				E		S	I	A	I	V		V	A	S			I	H	L							N	G		G			T	T		E	S					V	R	T			
Taiwan/N665/09	D				H	K					R				E		S	I	A	I	V		V	A	S			I	H	L							N	G		G			T	T		E	S					V	R	T			
Taiwan/M1157/09	D				H	K					R				E		S	I	A	I	V		V	A	S			I	H	L							N	G		G			T	T		E	S			S		V	R	T			
Taiwan/M1735/10	D				H	K					R				E		S	I	A	I	V		V	A	S			I	H	L							N	G		G			T	T		E	S					V	R	T			
Taiwan/N13327/10	D				H	K					R				E		S	I	A	I	V		V	A	S			I	H	L							N	G		G			T	T		E	S					V	R	T			N
Taiwan/M1074/10	D				H	K					R				E		S	I	A	I	V		V	A	S			I	H	L							N	G		G			T	T		E	S					V	R	T			
Taiwan/M1815/10	D				H	K			S		R				E		S	I	A	I	V		V	A	S			I	H	L							N	G		G			T	T		E	S					V	R	T			
Taiwan/M36/11	D				H	K					R				E		S	I	A	I	V		V	A	S			I	H	L							N	G		G			T	T		E	S					V	R	T			
Taiwan/N658/11	D				H	K					R				E		S	I	A	I	V		V	A	S			I	H	L							N	G		G			T	T		E	S					V	R	T			N
Taiwan/N15927/12	D				H	K					R				E		S	I	A	I	V		V	A	S			I	H	L							N	G		G			T	T		E	S					V	R	T			
Taiwan/M651/12	D				H	K					R				E		S	I	A	I	V		V	A	S			I	H	L							N	G		G			T	T		E	S					V	R	T			
Taiwan/M1188/12	D				H	K					R				E		S	I	A	I	V		V	A	S			I	H	L				S			N	G		G			T	T		E	S					V	R	T			
Taiwan/M725/13	D				H	K					R				E		S	I	A	I	V		V	A	S			I	H	L							N	G		G			T	T		E	S					V	R	T			
Taiwan/M807/13	D				H	K					R				E		S	I	A	I	V		V	A	S			I	H	L							N	G		G			T	T		E	S					V	R	T			
Taiwan/M977/13	D				H	K					R				E		S	I	A	I	V		V	A	S			I	H	L							N	G		G			T	T		E	S					V	R	T			
Taiwan/M593/13	D				H	K					R				E		S	I	A	I	V		V	A	S			I	H	L							N	G		G			T	T		E	S					V	R	T			
Taiwan/M216/14	D				H	K					R				E		S	I	A	I	V		V	A	S			I	H	L							N	G		G			T	T		E	S					V	R	T			
Taiwan/M133/14	D				H	K					R				E		S	I	A	I	V		V	A	S			I	H	L							N	G		G		K	T	T		E	S					V	R	T			
Taiwan/M202/15	D				H	K					R				E		S	I	A	I	V	G	V	A	S			I	H	L							N	G		G			T	T		E	S					V	R	T			
Taiwan/M513/15	D				H	K					R				E		S	I	A	I	V		V	A	S			I	H	L							N	G		G			T	T		E	S					V	R	T			
Taiwan/M1386/15	D				H	K					R				E		S	I	A	I	V		V	A	S			I	H	L							N	G		G			T	T		E	S					V	R				
Taiwan/28588/16	D				H	K					R				E		S	I	A	I	V		V	A	S			I	H	L							N	G		G			T	T		E	S					V	R				
Taiwan/M50671/16	D				H	K					R				E		S	I	A	I	V		V	A	S			I	H	L							N	G		G			T	T		E	S					V	R				
Taiwan/M50214/17	D				H	K					R				E		S	I	A	I	V		V	A	S			I	H	L							N	G		G			T	T		E	S					V	R				
Taiwan/M50339/17	D				H	K					R				E		S	I	A	I	V		V	A	S			I	H	L							N	G		G			T	T		E	S					V	R				
